# ELIXCYTE^®^, an Allogenic Adipose-Derived Stem Cell Product, Mitigates Osteoarthritis by Reducing Inflammation and Preventing Cartilage Degradation In Vitro

**DOI:** 10.3390/cimb46080495

**Published:** 2024-08-02

**Authors:** Yu-Hsiu Chen, Yi-Pei Hung, Chih-Ying Chen, Yi-Ting Chen, Tai-Chen Tsai, Jui-Jung Yang, Chia-Chun Wu

**Affiliations:** 1 Rheumatology/Immunology and Allergy, Department of Internal Medicine, Tri-Service General Hospital, National Defense Medical Center, Taipei 114202, Taiwan; tinachen70@mail.ndmctsgh.edu.tw; 2 UnicoCell Biomed Co., Ltd., Taipei 11494, Taiwan; yipeihung@unicocell.com (Y.-P.H.); amy.chen@unicocell.com (C.-Y.C.); yiting.chen@unicocell.com (Y.-T.C.);; 3 Department of Orthopedics, Tri-Service General Hospital, National Defense Medical Center, Taipei 114202, Taiwan; doc931012003@yahoo.com.tw

**Keywords:** adipose-derived stem cells, anti-inflammatory cytokines, ELIXCYTE^®^, normal human articular chondrocytes, osteoarthritis, synovial fluid

## Abstract

Adipose-derived stem cells (ADSCs) comprise a promising therapy for osteoarthritis (OA). The therapeutic potential of ELIXCYTE^®^, an allogeneic human ADSC (hADSC) product, was demonstrated in a phase I/II OA clinical trial. However, the exact mechanism underlying such effects is not clear. Moreover, studies suggest that interleukin-11 (IL-11) has anti-inflammatory, tissue-regenerative, and immune-regulatory functions. Our aim was to unravel the mechanism associated with the therapeutic effects of ELIXCYTE^®^ on OA and its relationship with IL-11. We cocultured ELIXCYTE^®^ with normal human articular chondrocytes (NHACs) in synovial fluid obtained from individuals with OA (OA-SF) to investigate its effect on chondrocyte matrix synthesis and degradation and inflammation by assessing gene expression and cytokine levels. NHACs exposed to OA-SF exhibited increased *MMP13* expression. However, coculturing ELIXCYTE^®^ with chondrocytes in OA-SF reduced *MMP13* expression in chondrocytes and downregulated *PTGS2* and *FGF2* expression in ELIXCYTE^®^. ELIXCYTE^®^ treatment elevated anti-inflammatory cytokine (IL-1RA, IL-10, and IL-13) levels, and the reduction in *MMP13* was positively correlated with IL-11 concentrations in OA-SF. These findings indicate that IL-11 in OA-SF might serve as a predictive biomarker for the ELIXCYTE^®^ treatment response in OA, emphasizing the therapeutic potential of ELIXCYTE^®^ to mitigate OA progression and provide insights into its immunomodulatory effects.

## 1. Introduction

Osteoarthritis (OA) is a prevalent degenerative joint disease characterized by inflammation and deterioration of the cartilage and bone structure [1]. The associated symptoms include pain, stiffness, limited joint motion, decreased mobility, and increased mortality among patients with OA [2]. Current pharmacological treatments for OA include analgesics, non-steroidal anti-inflammatory drugs, and intra-articular corticosteroids or hyaluronic acid injections to provide pain relief or anti-inflammatory effects [3]. In cases of severe OA or when conservative measures fail, joint replacement surgery is considered, and this involves the use of artificial implants to replace damaged joints [3].

Adipose-derived stem cells (ADSCs) have emerged as a promising and novel therapy for OA [4,5]. Studies have shown that the use of ADSCs in patients with OA can result in pain reduction and improved physical functions [5]. Moreover, the ability of ADSCs to reduce joint inflammation and improve clinical symptoms and radiographic changes associated with OA has been demonstrated [5]. ELIXCYTE^®^ is an allogeneic ADSC product developed by the Stem Cell Bank system of UnicoCell Biomed. Its allogeneic properties provide a readily available, minimally invasive, and cost-effective treatment option, which is particularly advantageous for the elderly, who are more likely to suffer from OA. ELIXCYTE^®^ has been evaluated for safety and efficacy in a phase I/II clinical trial (Identifier: NCT02784964), and the results indicated its therapeutic potential for knee OA [6]. Findings further suggest that ELIXCYTE^®^ might have anti-inflammatory properties and the ability to promote cartilage matrix synthesis [6]. However, the exact mechanism underlying this therapeutic effect remains unclear.

Synovial fluid (SF) collected from patients with OA (OA-SF) often exhibits elevated levels of pro-inflammatory cytokines and chemokines [7]. Prior studies have shown that OA-SF can induce the secretion of pro-inflammatory cytokines, including interleukin (IL)-6, IL-8, and MCP-1, in human primary chondrocytes [8]. Additionally, the inflamed SF has been shown to induce the release of IL-10, IL-1β, TNF-α, and IL-6 from bone marrow mononuclear cells in vitro [9]. These cytokines can potentially stimulate the expression of immunomodulatory genes in ADSCs [10]. Another study investigating the secretory profile of ADSCs treated with OA-SF identified various secretome components with roles in its protective effect on OA cartilage, ECM homeostasis, and MMP inhibition, and these include *IGFBP4*, *IGFBP6*, *TIMP1*, *TIMP2*, *uPA/PLAUR*, and *Serpine1* [11]. Recent studies also indicate that mesenchymal stem cells (MSCs) and IL-11 have anti-inflammatory, tissue-regenerative, and immune-regulatory functions and that they interact with IL-6 [12,13]. During normal homeostasis, IL-11 expression levels are typically low and challenging to detect. However, IL-11 expression was found to be increased with OA severity in the OA-SF patients [14].

In this study, we aimed to elucidate the effect of ELIXCYTE^®^ on inflammation and cartilage matrix dynamics within the context of OA pathology. To simulate the diverse nature of human OA, we mimicked an in vitro OA environment by introducing the SF obtained from patients with knee OA to primary normal human articular chondrocytes (NHACs). Subsequently, we conducted coculture experiments involving ELIXCYTE^®^ to gain insights into the mechanisms underlying its potential to elicit anti-inflammatory responses and promote chondrogenesis.

## 2. Materials and Methods

### 2.1. Synovial Fluid Collection

SF samples were collected from 12 patients diagnosed with OA, while they were affected by hydrarthrosis, with a mean age of 62.00 ± 6.91 years and a male-to-female ratio of 1:2. Among these samples, six were obtained from patients with knee OA classified as Kellgren–Lawrence (KL) grade II, whereas the remaining six samples were from patients with OA of KL grade III. All samples were obtained with IRB approval from Tri-Service General Hospital (IRB number: 1-108-05-144), and all patients consented to the use of their SF for scientific research purposes. After joint aspiration, a 20 mL synovial fluid specimen was collected and then centrifuged at 400× *g* for 10 min to remove the debris. The resulting supernatant was then aliquoted and stored at −80 °C until further use.

### 2.2. ELIXCYTE^®^ Isolation and Culture

ELIXCYTE^®^ (UnicoCell BioMed), an allogeneic ADSC product developed by UnicoCell Biomed Co., Ltd., Taipei, Taiwan, was obtained and isolated according to the methodology described in a previous study [6]. ELIXCYTE^®^ was stored in the UnicoCell Adipose Stem Cell Bank, which was certified through the U.S. FDA Master File (MF number: 28993), ensuring the long-term stability and consistency of biologics. Briefly, adipose tissue was collected using ultrasonic-assisted liposuction. The stromal vascular fraction was isolated by digesting the adipose tissue with type I and type II collagenase. The adherent cells with high proliferation capacity were selected as ELIXCYTE^®^, and they were subsequently detached and propagated to passages 4–7 for use in this experiment. For ELIXCYTE^®^ culture, the cells were thawed and cultured in α-MEM (Thermo Fisher Scientific, Waltham, MA, USA, Cat. 32561) supplemented with 5% human platelet lysate (hPL; AventaCell, Kent, WA, USA, Cat. HPCPLCRL50) in a humidified incubator with 5% CO_2_ at 37 °C.

### 2.3. Chondrocyte Cell Culture

Human chondrocytes, specifically Clonetics™ Normal Human Articular Chondrocytes (NHACs; Lonza, Basilea, Switzerland, Cat. CC-2550), were purchased and cultured in Chondrocyte Medium (ScienCell, San Diego, CA, USA, Cat. 4651) in a humidified incubator with 5% CO_2_ at 37 °C. The NHACs were primary chondrocytes isolated from human knee tissue, sub-cultured to passage 4, and used for all the experiments.

### 2.4. Coculture of NHACs with ELIXCYTE^®^ in an OA-SF Environment

**Figure 1:**
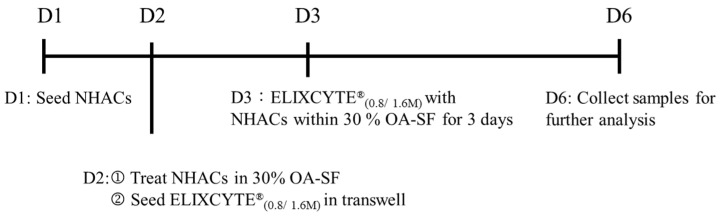
Timeline of the coculture systems. On day 1, NHAC were seeded onto culture plates and allowed to attach. The following day, NHACs were treated with a 30% dilution of SF obtained from patients with OA. Simultaneously, ELIXCYTE^®^ was seeded in a transwell plate. On day 3, NHACs and ELIXCYTE^®^ were cocultured together within a 30% dilution of OA-SF, facilitating their interaction in the shared environment. Finally, cell pellets were collected for further analysis.

To evaluate the effects of ELIXCYTE^®^ on NHACs within the OA-SF environment, the following process was performed. NHACs were trypsinized at 70% confluency, seeded at a density of 1 × 10^5^ cells per well in a 6-well plate with chondrocyte medium, and allowed to adhere overnight. The following day, the medium was collected, and the cells were washed once with PBS. NHACs were then treated with a 30% dilution of SF in α-MEM supplemented with 1% fetal bovine serum for 1 day, producing NHACs cultured in OA-SF, designated as NHAC_SF_. Concurrently, ELIXCYTE^®^ cells were seeded at a density of 0.8 × 10^6^ (0.8 M) or 1.6 × 10^6^ (1.6 M) cells in a 6-well transwell plate (Corning, NY, USA, Cat. 3450) and cultured in α-MEM supplemented with 5% hPL for 1 day. Following incubation, the culture media were collected for further analysis. Furthermore, ELIXCYTE^®^ (0.8 M or 1.6 M) was cocultured with NHACs in the OA-SF for 3 days. On the final day, the culture media were collected, and both NHAC_coculture (0.8M or 1.6M)_ and ELIXCYTE^®^_coculture (0.8M or 1.6M)_ samples were harvested and stored at −80 °C for future analysis. The coculture process is illustrated in Figure 1.

### 2.5. Gene Expression Analysis

We assessed the gene expression levels of *COL2A1*, *ACAN*, and *MMP13* in NHACs and *IDO1*, *PTGS2*, *IL10*, *TGFB1*, *FGF1*, and *FGF2* in ELIXCYTE^®^. The total RNA was isolated using TRIzol reagent (Thermo Fisher Scientific, Waltham, MA, USA, Cat. 15596026) following the manufacturer’s instructions. Then, 2 µg of total RNA was used to synthesize the first-strand cDNA using Illustra^TM^ Ready-to-Go^TM^ RT-PCR beads (GE Healthcare, Chicago, IL, USA, Cat. 27925901). The qPCR process was performed using Fast SYBR green master mix (Thermo Fisher Scientific, Waltham, MA, USA, Cat. 4385612). *COL2A1*, *ACAN*, and *MMP13* are genes associated with chondrogenesis and matrix degradation [15]. *IDO1*, *PTGS2*, *IL10*, and *TGFB1* are genes associated with immune modulation [16], and *FGF1* and *FGF2* are related to cartilage matrix homeostasis and regulation [17,18]. *GAPDH* was employed as an internal control. The changes in the expression levels of differentially expressed RNAs were calculated using the delta Ct value (ΔCt = Ct_test_ − Ct_GAPDH_). The qPCR primers used in this study are listed in Table S1.

### 2.6. Multiplexed Arrays

The levels of cytokines, chemokines, and growth factors were determined in all cell culture supernatants collected. The compositions of these samples were analyzed using the MILLIPLEX^®^ Human Cytokine/Chemokine/Growth Factor Panel A (Merck, Darmstadt, Germany, Cat. HCYTA-60K) to identify and quantify the proteins present. The following proteins were examined in this study: IFN-γ, IL-1β, IL-1RA, IL-6, IL-8, IL-10, IL-12p70, IL-13, IL-17A, IP-10, MCP-1, MIP-1α, TNF-α, and VEGF-A. All measurements were performed using a MAGPIX instrument according to the manufacturer’s instructions. 

### 2.7. IL-11 ELISA

The concentration of IL-11 in OA-SF was analyzed using a Human IL-11 Quantikine ELISA kit (R&D Systems, Minneapolis, MN, USA, Cat. D1100). Prior to analysis, SFs were pretreated with 20 mg/mL of hyaluronidase (Sigma-Aldrich, St. Louis, MO, USA, Cat. SI-H3506) for 15 min at room temperature.

### 2.8. Data Analysis

The statistical analyses were conducted using GraphPad Prism 9 software (Dotmatics, Bishops Stortford, UK). The data are presented as the mean ± standard deviation (SD). Independent *t*-tests were performed to compare NHACs vs. NHAC_SF_. Paired *t*-tests were performed for the supernatant of NHAC_SF_ vs. coculture_1.6M_ comparison. A one-way ANOVA with Dunnett’s post hoc test was used to analyze group differences. Pearson correlation analysis was employed to examine the relationship between the reduction in *MMP13* levels mediated by ELIXCYTE^®^ (ΔΔCt = ΔCt of NHAC_coculture_ − ΔCt of NHAC_SF_) and IL-11 concentrations in the 12 OA-SF samples. A *p*-value less than 0.05 was considered statistically significant.

## 3. Results

### 3.1. OA-SF Upregulates MMP13 Gene Expression in Chondrocytes, whereas Coculture with ELIXCYTE^®^ Downregulates MMP13 Expression

**Figure 2:**
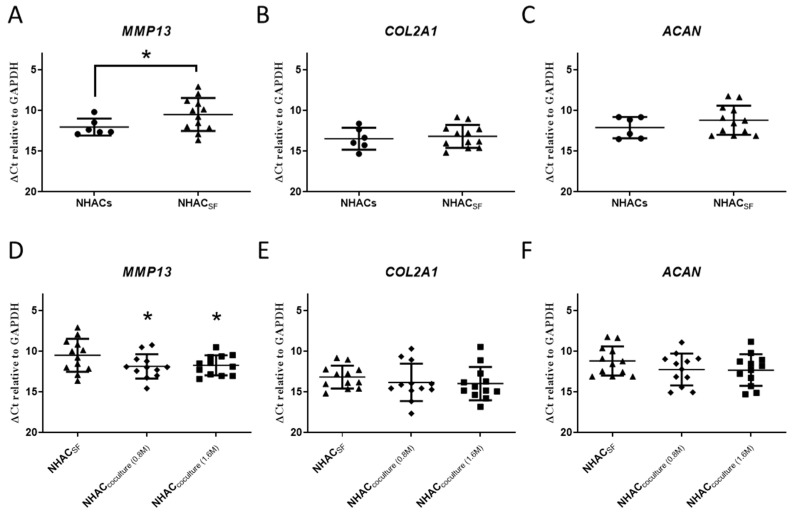
Gene expression of ELIXCYTE^®^ on NHACs in an environment. (**a–c**) Comparison of the gene expression levels of *MMP13* (**A**), *COL2A1* (**B**), and *ACAN* (**C**) between NHACs (n = 6) and NHAC_SF_ (n = 12). (**D**–**F**) Analysis of the gene expression levels of *MMP13* (**D**), *COL2A1* (**E**), and *ACAN* (**F**) between NHAC_SF_, NHAC_coculture-0.8M_, and NHAC_coculture-1.6M_ (n = 12, paired). The gene expression levels, measured as delta Ct (∆Ct) values relative to the *GAPDH* reference gene, are presented in a dot graph format with the mean ± SD. Independent *t*-tests were performed to compare NHACs and NHAC_SF_. Repeated-measures ANOVA with Dunnett’s post hoc test was performed to compare NHAC_SF_, NHAC_coculture-0.8M_, and NHAC_coculture-1.6M_ (*, *p* < 0.05).

The mean gene expression (ΔCt relative to *GAPDH*) of *MMP13* was significantly higher in NHACs in the presence of OA-SF (NHAC_SF_) than in NHACs (10.49 ± 2.02 vs. 12.03 ± 1.04, *p* < 0.05), corresponding to a mean 2.9-fold change (Figure 2A). However, there were no significant differences in the mean gene expression of *COL2A1* and *ACAN* between NHACs and NHAC_SF_ (Figure 2B,C). Our findings indicate that OA-SF increased expression of the cartilage-degradation marker *MMP13* but did not affect chondrogenesis in chondrocytes. Next, we evaluated the effect of ELIXCYTE^®^ on NHACs within an OA-SF environment. We found that the mean gene expression (ΔCt relative to *GAPDH*) of *MMP13* was significantly reduced in NHAC_coculture-0.8M_ and NHAC_coculture-1.6M_ compared to that in NHAC_SF_ (*p* < 0.05; Figure 2D). Moreover, the mean gene expression of *MMP13* in NHAC_coculture-0.8M_, NHAC_coculture-1.6M_, and NHAC_SF_ was 11.85 ± 1.50, 11.72 ± 1.23, and 10.49 ± 2.02, respectively, indicating a 0.39-fold change and 0.43-fold change compared to the expression in NHAC_SF_. However, no significant differences in the expression of *COL2A1* and *ACAN* were observed between NHAC_SF_, NHAC_coculture-0.8M_, and NHAC_coculture-1.6M_ (Figure 2E,F). Overall, our findings suggest that exposing NHACs to OA-SF increases the expression of *MMP13*. In addition, both 0.8 M and 1.6 M ELIXCYTE^®^ had similar effects on reducing *MMP13* gene expression, while no dosage-dependent effect was observed. This suggests that ELIXCYTE^®^ could mitigate the detrimental effects of OA-SF on cartilage degradation.

### 3.2. ELIXCYTE^®^ Protects NHACs against the Effects of OA-SF through Anti-Inflammatory Activity

**Figure 3:**
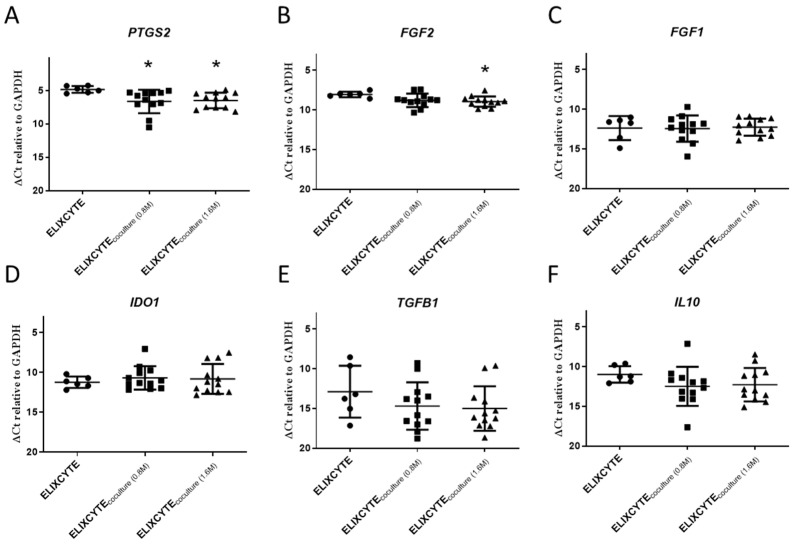
Gene expression of coculturing NHAC within OA-SF on ELIXCYTE^®^. The gene expression levels of *PTGS2* (**A**), *FGF2* (**B**), *FGF1* (**C**), *IDO1* (**D**), *TGFB1* (**E**), and *IL10* (**F**) of ELIXCYTE^®^ were quantified by qPCR. Different doses (0.8 M or 1.6 M) of ELIXCYTE^®^ coculture were used in the NHAC_SF_ environment, compared with ELIXCYTE^®^. GAPDH was used as the internal control. Results are represented as mean ± SD. For statistical analysis, an ANOVA test with Dunnett’s post hoc test was used (*, *p* < 0.05).

The expression of *PTGS2*, which encodes a pro-inflammatory factor, was significantly downregulated in ELIXCYTE^®^_coculture-0.8M_ and ELIXCYTE^®^_coculture-1.6M_ (Figure 3A). The mean gene expression (ΔCT relative to *GAPDH*) of *PTGS2* in ELIXCYTE^®^_coculture-0.8M_, ELIXCYTE^®^_coculture-1.6M_, and ELIXCYTE^®^ groups was 6.63 ± 1.41, 6.49 ± 1.17, and 4.83 ± 0.5, respectively, corresponding to a 0.29-fold change and 0.32-fold change (*p* < 0.05). This indicates that ELIXCYTE^®^ can reduce the expression of inflammatory factors in a pro-inflammatory environment. For matrix-degeneration-related genes, *FGF2* was reduced in the ELIXCYTE^®^_coculture-1.6M_ group, and the mean gene expression (ΔCt relative to *GAPDH*) of *FGF2* in ELIXCYTE^®^ and ELIXCYTE^®^_coculture-1.6M_ groups was 8.04 ± 0.35 and 9.02 ± 0.67 (Figure 3B). However, we did not observe significant changes in *FGF1*, *IDO1*, *IL10*, and *TGFB1* between ELIXCYTE^®^, ELIXCYTE^®^_coculture-0.8M_, and ELIXCYTE^®^_coculture-1.6M_ groups (Figure 3C–F). Our finding suggests that ELIXCYTE^®^ has anti-inflammatory activity with potential effects on matrix degeneration through a reduction in *PTGS2* and *FGF2* expression.

### 3.3. ELIXCYTE^®^ Triggers Anti-Inflammatory and Pro-Inflammatory Factors in the Coculture System

**Figure 4:**
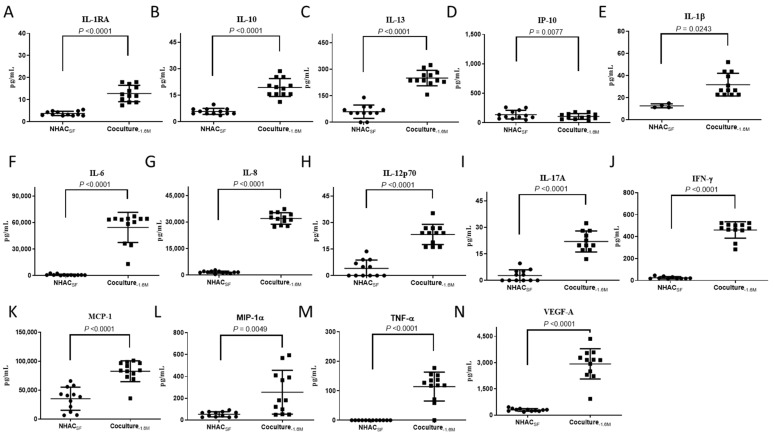
Levels of various cytokines, chemokines, and growth factors in the supernatant of NHAC_SF_ (NHACs within OA-SF) without and with ELIXCYTE^®^ coculture. The levels of IL-1RA (**A**), IL-10 (**B**), IL-13 (**C**), IP-10 (**D**), IL-1β (**E**), IL-6 (**F**), IL-8 (**G**), IL-12p70 (**H**), IL-17A (**I**), IFN-γ (**J**), MCP-1 (**K**), MIP-1α (**L**), TNF-α (**M**), and VEGF-A (**N**) were measured. The data are presented as the mean ± standard deviation (SD) from a paired sample size of n = 12.

We further conducted a protein array analysis to identify the cytokines involved in the composition of SF and their changes before and after treatment with ELIXCYTE^®^. We analyzed 14 proteins, including IFN-γ, IL-1β, IL-1RA, IL-6, IL-8, IL-10, IL-12p70, IL-13, IL-17A, IP-10, MCP-1, MIP-1α, TNF-α, and VEGF-A. The protein levels of these cytokines/chemokines varied after ELIXCYTE^®^ treatment. For the natural anti-inflammatory factors, including IL-1RA, IL-10, and IL-13, protein levels were higher in the coculture_-1.6M_ media than in the NHAC_SF_ media (Figure 4A–C). Moreover, the protein level of IP-10, a chemokine related to inflammation and immune cell recruitment, was decreased in the coculture_-1.6M_ media compared to that in the NHAC_SF_ media (Figure 4D). In contrast, for the pro-inflammatory cytokines, including IFN-γ, IL-1β, IL-6, IL-8, IL-12p70, IL-17A, MCP-1, MIP-1α, TNF-α, and VEGF-A, protein expression was also elevated in the coculture_-1.6M_ media (Figure 4E–N). However, the baseline levels of IFN-γ, IL-1β, IL-6, IL-8, IL-12p70, IL-17A, MCP-1, MIP-1α, TNF-α, and VEGF-A were higher in the ELIXCYTE^®^ media than with NHACs and NHAC_SF_ (Figure S1). Overall, the protein array analysis revealed both increases and decreases in the protein levels of various cytokines/chemokines in the media after coculture with ELIXCYTE^®^. These findings suggest that ELIXCYTE^®^ has the potential to influence the cytokine profile and inflammatory environment of OA, possibly contributing to immunomodulatory effects in the context of OA.

### 3.4. IL-11 in OA-SF Is Positively Correlated with a Reduction in MMP13 Levels

**Figure 5:**
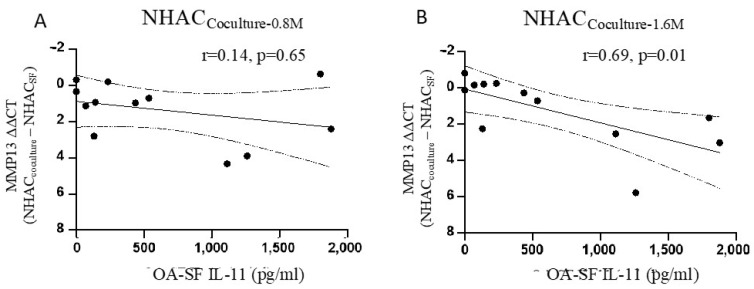
Correlation between the reduction in *MMP13* expression in NHACs after coculture with 0.8 M (**A**) and 1.6 M (**B**) of ELIXCYTE^®^ and the concentration of IL-11 in OA-SF. The X-axis represents the IL-11 concentrations, and the Y-axis represents the effects of ELIXCYTE^®^ treatment on *MMP13* gene expression (gene expression for NHAC_Coculture_ − gene expression for NHACSF). The correlation between these two variables was assessed by performing Pearson correlation analysis (n = 12). The dot plot with a regression line demonstrates the positive correlation between OA-SF IL-11 and reduction of *MMP13* expression, The dashed lines represent the 95% confidence interval.

Furthermore, we investigated the association between the concentration of IL-11 in OA-SF and the reduction in *MMP13* gene expression of NHACs before and after coculture with ELIXCYTE^®^ in an OA-SF environment. We found a positive correlation between the IL-11 concentration in OA-SF and a reduction in *MMP13* expression (ΔΔCt), with an r-value of 0.69 (*p* = 0.01), after 1.6 M ELIXCYTE^®^ treatment (Figure 5A), but not with 0.8 M ELIXCYTE^®^ (Figure 5B). This suggests that IL-11 might play a role in mediating the reduction in *MMP13* expression in NHACs through an interaction with ELIXCYTE^®^.

## 4. Discussion

In this study, we demonstrated that the gene expression of *MMP13*, encoding a matrix-degrading enzyme associated with cartilage degradation, was increased in NHACs stimulated by OA-SF. However, coculture with ELIXCYTE^®^ was able to inhibit the upregulation of *MMP13* gene expression induced by OA-SF. Moreover, reduced expression of *PTGS2* and *FGF2* was observed in the ELIXCYTE^®^_coculture_ group compared to that in the ELIXCYTE^®^ group, suggesting the anti-inflammatory properties of ELIXCYTE^®^ and its potential effect on matrix degeneration. Furthermore, we found higher levels of anti-inflammatory cytokines, including IL-1RA, IL-10, and IL-13, and decreased pro-inflammatory chemokines, namely IP-10, in the coculture media. Moreover, IL-11 levels showed a positive association with *MMP13* reduction in OA-SF, suggesting that IL-11 could be a predictive biomarker of favorable responses to ELIXCYTE^®^ in OA. Collectively, our findings suggest that ELIXCYTE^®^ has the potential to modulate gene expression, inhibit cartilage degradation markers, and exhibit anti-inflammatory properties, and that it might be influenced by the presence of IL-11 when predicting favorable responses in OA.

Previous research has shown that SF obtained from patients with OA can induce the production of pro-inflammatory cytokines, decrease chondrogenic markers, and increase the expression of *MMP3* and *MMP13* in chondrocytes [8,19,20]. Our result is consistent with these previous studies, indicating that *MMP13* expression is upregulated in NHACs after culture in OA-SF, which suggests that we successfully simulated an OA environment. Moreover, we found that ELIXCYTE^®^ coculture with NHAC_SF_ decreased *MMP13* gene expression. This suggests that ELIXCYTE^®^ can reduce the expression of *MMP13* in NHACs, thereby potentially protecting chondrocytes from injury within the OA environment. There were no statistically significant differences in expression levels of *COL2A1* and *ACAN* in the NHAC_coculture_ group, which was related to the chondrogenesis properties of chondrocytes, compared to that in the NHAC_SF_ group. Our results indicate that ELIXCYTE^®^ may protect against OA by attenuating inflammation-mediated cartilage degradation but may not promote the synthesis of the cartilage matrix.

A reduction in *PTGS2* and *FGF2* expression was observed in the ELIXCYTE^®^_coculture_ group compared to that in the ELIXCYTE^®^ group. *PTGS2*, also known as COX-2, plays a critical role in the inflammatory process, and its increased expression is associated with pain sensations during acute inflammation [21]. Therefore, selective COX-2 inhibitors are commonly used to treat OA to mitigate pain and inflammation [22]. Our findings suggest that ELIXCYTE^®^, when cocultured with NHAC in OA-SF environment, effectively decreases the pro-inflammatory marker *PTGS2* (COX-2) and exhibits anti-inflammatory properties. In addition to its anti-inflammatory effects, ELIXCYTE^®^ influences cartilage matrix-degradation-related genes, particularly *FGF2*. *FGF2* is known to stimulate *MMP13* expression in osteoblasts, chondrosarcoma cells, and articular chondrocytes [23,24,25], and it can upregulate the expression of *MMP1* and *MMP13*, promoting matrix degradation through neuro-endocrine pathways in human articular chondrocytes [26]. This upregulation of MMP expression is crucial for tissue remodeling and OA progression [27]. Our study observed a reduction in *FGF2* gene expression in the ELIXCYTE^®^_coculture_ group compared to ELIXCYTE^®^ alone, suggesting that ELIXCYTE^®^ may regulate *FGF2* expression and its downstream effects on MMPs, thereby potentially mitigating cartilage degradation. These findings provide insights into the multifaceted interactions among OA-SF, chondrocytes, and ELIXCYTE^®^, highlighting the modulation of inflammation through *PTGS2* (COX-2) and the regulation of matrix-degradation-related genes through *FGF2*. This complex interplay underscores the potential therapeutic role of ELIXCYTE^®^ in managing OA by targeting both inflammatory and degradative pathways.

In prior studies, elevated levels of pro-inflammatory chemokines, cytokines, and growth factors in OA-SF were associated with chronic inflammation, including IFN-γ, IL-1β, IL-6, IL-8, MCP-1, TNF-α, and VEGF levels [28]. Further, MSCs exert immunomodulatory effects by suppressing the expression of various inflammatory cytokines, including TNF-α, IL-1β, IL-3, IL-7, IL-8, IL-17, IL-21, IL-22, and IFN-γ, while simultaneously increasing the expression of anti-inflammatory cytokines, such as IL-4 and IL-10 [29,30]. Additionally, MSCs are known to secrete immunosuppressive factors and anti-inflammatory factors, such as TGF-β, VEGF, IL-10, IL-12p70, IL-13, PGE2, and IDO, to modulate the mechanisms underlying disease initiation and progression in the body [31]. Here, we reported higher levels of anti-inflammatory cytokines, including IL-1RA, IL-10, and IL-13, and decreases in pro-inflammatory chemokines, such as IP-10, in the coculture media. This reduction in IP-10 levels is consistent with that reported in previous studies, which have shown the potential role of IP-10 in OA pathogenesis, including its involvement in neutrophil recruitment, natural killer cell activation, and pain sensation in patients with OA [24,25,26]. Our findings suggest that ELIXCYTE^®^ promotes the production or accumulation of these anti-inflammatory cytokines, potentially contributing to the attenuation of inflammation.

IL-11, a member of the IL-6 family of cytokines, plays a multifaceted role in different processes, such as hematopoiesis, bone formation, tissue regeneration, inflammation, and tumor progression [32,33]. IL-11 can induce aggrecanase activity, leading to cartilage damage in inflammatory arthritis [34]. In our previous studies, we demonstrated that IL-11 is expressed at higher levels in damaged cartilage and serves as a predictive factor for positive responses to conventional treatments in OA [35,36]. Here, we found a correlation between the concentration of IL-11 in OA-SF and the suppressive effect of ELIXCYTE^®^ on *MMP13* expression in NHACs. Collectively, these findings suggest that IL-11 might have a role in modulating the response of chondrocytes to ELIXCYTE^®^ treatment. This finding indicates that IL-11 may be a viable therapeutic target in the context of OA and that it has potential utility as a predictive factor when assessing the effectiveness of ELIXCYTE^®^ treatment.

Our study had several strengths. ELIXCYTE^®^, an allogeneic ADSC product, offers the advantage of being easily produced, and it can be readily administered to elderly individuals who are more susceptible to the effects of OA. One notable strength of our approach is the use of synovial fluid obtained directly from patients with OA in our in vitro study. Unlike the use of drug-induced in vitro OA models, this choice enabled us to better recapitulate the diverse and heterogeneous nature of OA as it is manifested in different individuals. Furthermore, in addition to investigating the underlying mechanisms, we found that the IL-11 concentration in the synovial fluid might serve as a potential predictive biomarker for assessing the efficacy of ELIXCYTE^®^ treatment. However, there are some limitations to our study; first, the sample size, specifically 12 patients, used in this study might be considered small, which could affect the generalizability of the results. To enhance the robustness and reliability of the findings, future studies should consider larger sample sizes encompassing a diverse range of patients. Second, the study primarily relied on gene expression analysis and protein profiling, which provide valuable information but might not fully capture the functional implications of the observed changes. Incorporating functional assays to assess cellular behaviors, such as proliferation, differentiation, and matrix synthesis, would augment our understanding of the therapeutic potential of ELIXCYTE^®^. Moreover, this study focused solely on the interaction between ELIXCYTE^®^ and chondrocytes, neglecting the potential influence of other cell types present in the OA joint, such as synovial or immune cells. Exploring the broader cellular interactions within the OA joint would offer a more comprehensive understanding of the effects of ELIXCYTE^®^.

## 5. Conclusions

**Figure 6:**
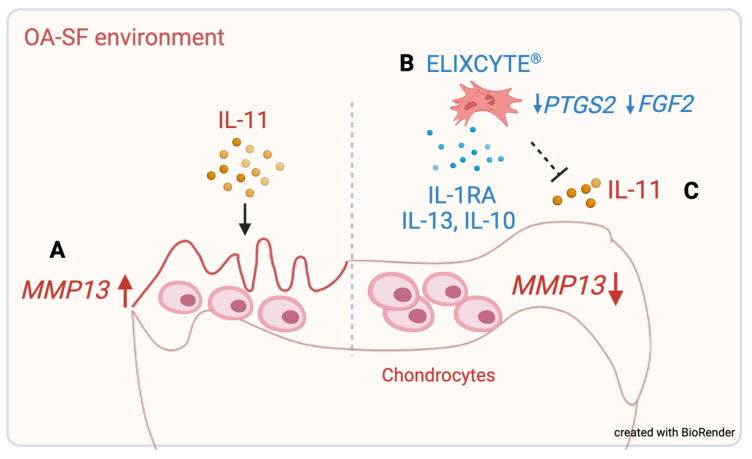
Graphic summary of results. (**A**) OA-SF increased *MMP13* expression in NHACs, while ELIXCYTE^®^ could mitigate cartilage degradation by reducing the expression of *MMP13*. (**B**) ELIXCYTE^®^ exhibited anti-inflammatory and matrix-preserving properties through its ability to downregulate the expression of PTGS2 and FGF2 and modulate cytokine profiles in the OA-SF environment. (**C**) ELIXCYTE^®^ might mediate the reduction in *MMP13* expression in NHACs through an interaction with IL-11.

This study provided insights into the mechanism underlying the potential therapeutic effects of ELIXCYTE^®^ in the context of OA. We found OA-SF increased *MMP13* expression in NHAC, while ELIXCYTE^®^ could mitigate cartilage degradation by reducing the expression of *MMP13*, a marker associated with matrix degradation (Figure 6A). Additionally, ELIXCYTE^®^ exhibited anti-inflammatory and matrix-preserving properties through its ability to downregulate the expression of *PTGS2* and *FGF2* and modulate cytokine profiles in the OA environment (Figure 6B). Moreover, IL-11 levels were associated with MMP13 reduction in OA-SF, highlighting the potential role of IL-11 as a predictive factor for favorable responses to ELIXCYTE^®^ treatment (Figure 6C).

## Data Availability

The data that support the findings of this study are available upon reasonable request from the corresponding author.

## Supplementary Materials

The following supporting information can be downloaded at https://www.mdpi.com/article/10.3390/cimb46080495/s1. Figure S1: Release of cytokines/chemokines/growth factors among different condition media. Table S1: Primer sequence used in the study.
