# Supplementary material for: ELIXCYTE®, an Allogenic Adipose-Derived Stem Cell Product, Mitigates Osteoarthritis by Reducing Inflammation and Preventing Cartilage Degradation In Vitro
Source: Curr Issues Mol Biol. 2024 Aug 2;46(8):8395–406. doi: 10.3390/cimb46080495 (PMC11352429; doi:10.3390/cimb46080495)
Supplement: Supplementary file 1 [file cimb-46-00495-s001.zip › cimb-3086360-supplementary.pdf]

ELIXCYTE®, an allogenic adipose-derived stem cell product, mitigates osteoarthritis by reducing inflammation and preventing cartilage degradation in vitro

Yu-Hsiu Chen [1], Yi-Pei Hung [2], Chih-Ying Chen [2], Yi-Ting Chen [2], Tai-Chen Tsai [2], Jui-Jung Yang [3] and Chia-Chun Wu [3,*]

SUPPLEMENTARY MATERIALS

Supplementary Table

Table S1. Primer sequence used in the study.

Gene name	Forward primer	Reverse primer
GAPDH	CACATGGCCTCCAAGGAGTAA	TGAGGGTCTCTCTCTTCCTCTTGT
COL2A1	ACTGGATTGACCCCAACCAA	TCCATGTTGCAGAAAACCTTCA
ACAN	TGCATTCCACGAAGCTAACCT	GACGCCTCGCCTTCTTGA
MMP13	TGTTGCTGCGCATGAGTTC	TGCTCCAGGGTCCTTGGA
IDO1	GCCTGATCTCATAGAGTCTGGC	TGCATCCCAGAACTAGACGTGC
PTGS2	AGGGTTGCTGGTGGTAGGAA	GGTCAATGGAAGCCTGTGATACT
IL10	TGAGAACAGCTGCACCCACTT	TCGGAGATCTCGAAGCATGTTA
TGFB1	TACCTGAACCCGTGTTGCTCTC	GTTGCTGAGGTATCGCCAGGAA
FGF1	ATGGCACAGTGGATGGGACAAG	TAAAAGCCCGTCGGTGTCCATG
FGF2	AGCGGCTGTACTGCAAAAACGG	CCTTTGATAGACACAACTCCTCTC

Supplementary Figures.

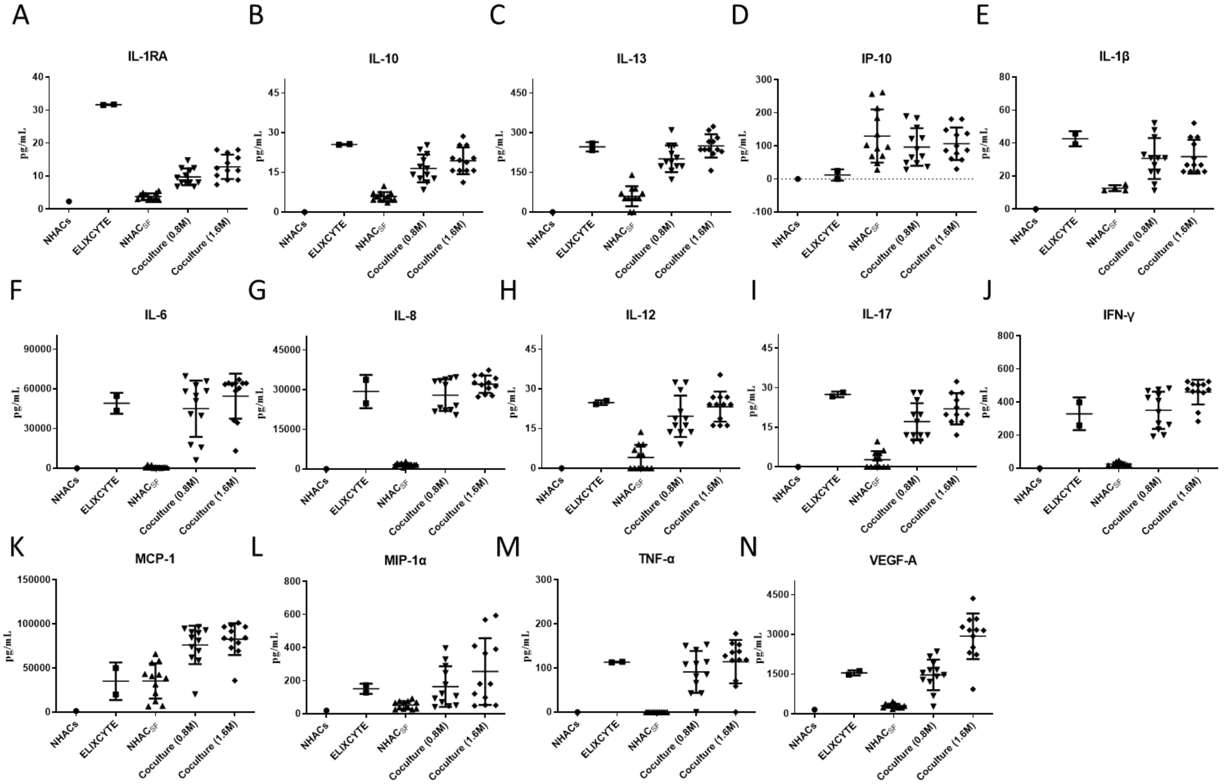

Figure S1. Release of cytokines/chemokines/growth factors among different condition media.

To observe the change of cytokines/chemokines/growth factors, we designed 6 groups, including NHACs, ELIXCYTE®, $NHAC_{SF}$, Coculture (0.8M) and Coculture (1.6M). (A–N) The secretion of IL-1RA (A), IL-10 (B), IL-13 (C), IP-10 (D), IL-1β (E), IL-6 (F), IL-8 (G), IL-12p70 (H), IL-17A (I), IFNγ (J), MCP-1 (K), MIP-1α (L), TNF-α (M), and VEGF-A (N) in supernatant of each group was detected. Data are presented as mean ± SD.
